# Prostate Cancer Diagnosis via Visual Representation of Tabular Data and Deep Transfer Learning

**DOI:** 10.3390/bioengineering11070635

**Published:** 2024-06-21

**Authors:** Moumen El-Melegy, Ahmed Mamdouh, Samia Ali, Mohamed Badawy, Mohamed Abou El-Ghar, Norah Saleh Alghamdi, Ayman El-Baz

**Affiliations:** 1 Electrical Engineering Department, Assiut University, Assiut 71516, Egypt; moumen@aun.edu.eg (M.E.-M.); ahmed.mamdouh@aun.edu.eg (A.M.); samia_fattah@aun.edu.eg (S.A.); 2 Radiology Department, Urology and Nephrology Center, Mansoura University, Mansoura 35516, Egypt; mohammed.ali.badawy@gmail.com (M.B.); maboelghar@mans.edu.eg (M.A.E.-G.); 3 Department of Computer Sciences, College of Computer and Information Sciences, Princess Nourah Bint Abdulrahman University, P.O. Box 84428, Riyadh 11671, Saudi Arabia; nosalghamdi@pnu.edu.sa; 4 Bioengineering Department, University of Louisville, Louisville, KY 40292, USA

**Keywords:** deep learning, machine learning, prostate cancer, stacking classifier, transfer learning

## Abstract

Prostate cancer (PC) is a prevalent and potentially fatal form of cancer that affects men globally. However, the existing diagnostic methods, such as biopsies or digital rectal examination (DRE), have limitations in terms of invasiveness, cost, and accuracy. This study proposes a novel machine learning approach for the diagnosis of PC by leveraging clinical biomarkers and personalized questionnaires. In our research, we explore various machine learning methods, including traditional, tree-based, and advanced tabular deep learning methods, to analyze tabular data related to PC. Additionally, we introduce the novel utilization of convolutional neural networks (CNNs) and transfer learning, which have been predominantly applied in image-related tasks, for handling tabular data after being transformed to proper graphical representations via our proposed Tab2Visual modeling framework. Furthermore, we investigate leveraging the prediction accuracy further by constructing ensemble models. An experimental evaluation of our proposed approach demonstrates its effectiveness in achieving superior performance attaining an F1-score of 0.907 and an AUC of 0.911. This offers promising potential for the accurate detection of PC without the reliance on invasive and high-cost procedures.

## 1. Introduction

Prostate cancer (PC) is a prevalent and potentially fatal cancer that affects men globally, and it is the second leading cause of cancer-related deaths in men worldwide after leukemia [1]. In the United States, approximately one in six men are diagnosed with PC [2]. The early detection of PC is crucial to increase the chances of successful treatment. The examination of a man’s blood for prostate-specific antigen (PSA) levels or via a digital rectal exam (DRE) are common routines for PC diagnosis. If abnormal results are obtained from either of these tests, a prostate biopsy is recommended to assess the prognosis of individuals with PC [3]. However, these tests have limitations in terms of accuracy and invasiveness. The PSA test has a high incidence of false positives and false negatives [4], while DRE and prostate biopsies, particularly the latter, are associated with a high degree of invasiveness and have the potential to induce serious physical harm [5,6]. As such, current diagnostic methods need improvement to reduce the risk of harm and to increase accuracy. The development of accurate, low-cost, and non-invasive diagnostic models has thus become a crucial area of research.

Over the past few years, several studies have highlighted the potential of machine learning in detecting prostate cancer at an early stage. For example, Wang et al. [7] conducted a study where they developed a machine learning approach for predicting PC using radiomic features extracted from multiparametric magnetic resonance imaging (MRI) data. The model demonstrated a high level of accuracy, achieving an area under the curve (AUC) of 0.905. In a separate investigation, Song et al. [8] developed a machine learning model based on convolutional neural networks (CNNs) to detect PC by analyzing histopathological images of biopsy samples. The model outperformed traditional diagnostic methods, achieving an AUC of 0.937. In another study, Varghese et al. [9] proposed a deep learning model for predicting the aggressiveness of PC using clinical and histopathological features. They achieved an accuracy of 87.7% in predicting the presence of high-grade PC. In a recent study, Peng et al. [10] introduced a machine learning approach for predicting the presence of clinically significant prostate cancer (CSPC) using clinical and imaging features. The study employed a deep learning model that integrated CNNs with gradient boosting machine (GBM) [11]. On the validation set, the model achieved an AUC of 0.88. The findings of the study suggested that machine learning models had the potential to accurately predict the diagnosis of CSPC in a non-invasive manner, thereby reducing unnecessary biopsies. Bhattacharya et al. [12] proposed a radiogenomic approach to predict the prognosis of prostate cancer. The researchers combined radiomic features extracted from MRI images with genetic data to develop their model. The results showed that the model achieved a higher accuracy in predicting PC recurrence compared to traditional clinical methods.

In a study focused on detecting prostate cancer from pre-biopsy information [13], various machine learning techniques, including artificial neural networks (ANNs), random forests (RF), and support vector machines (SVMs), were employed. The study’s results indicated that these methods achieved relatively high F1-score and AUC when classifying samples into two categories (benign/insignificant cancer vs. significant cancer). However, the same methods achieved lower F1-scores and AUC values when classifying samples into three categories (benign, insignificant, and significant). These findings suggest that machine learning techniques may be effective in detecting prostate cancer, but their performance may vary depending on the specific classification task. Furthermore, Perera et al. [14] utilized a four-layer neural network to detect prostate cancer using PSA and age information, achieving an AUC value of 0.72. Lee et al. [15] used the Synthetic Minority Oversampling Technique (SMOTE) [16] to balance the data used for predicting prostate cancer from PSA, DRE, and age information. The authors explored the application of RFs, SVMs, logistic regression (LR), extreme gradient boosting (XGBoost) [17], and light gradient boosted machine (LightGBM) [18]. RFs outperformed the other methods for patients aged 75 or older, while LR yielded better results for patients below the age of 75.

Very recently, some researchers have considered multi-omics data for enhancing prostate cancer diagnosis. Two notable examples are [19,20]. The first work relies on uniform manifold approximation and projection (UMAP) to embed gene expression, DNA methylation, and copy number alteration to create images used later for classification by CNNs. The latter work utilizes the Pairwise Controlled Manifold Approximation Projection (PaCMAP) dimensionality reduction technique to embed various omic sources in a CNN prediction model for PC grading.

Upon conducting a thorough analysis of the literature, the following becomes apparent:

- Radiographic and imaging-based diagnostic methods have shown high capabilities in detecting prostate cancer. However, these approaches often come at a high cost. Conversely, invasive procedures like biopsies raise concerns regarding potential harm to patients.
- Several of the aforementioned methods aim to reduce the need for invasive biopsies in prostate cancer diagnosis by utilizing machine learning techniques that rely on clinical biomarkers. These techniques utilize structured tabular data as input, which is easier and cheaper to obtain than medical images. Tabular data exhibit heterogeneity among their features, resulting in weaker correlations among them compared to homogeneous data, such as images or text. Tabular data often involve numerical and categorical features, where numerical features tend to have more dense representations, while categorical features tend to have more sparse representations. It thus becomes challenging to identify and exploit correlations among features without relying on spatial information.
- In classification problems involving tabular data, tree-based methods are frequently employed. These methods construct a hierarchical structure resembling a tree to make predictions about the target variable class using the available features. They are popular due to their ability to efficiently select relevant features and provide statistical information gains. Moreover, they offer advantages, such as quick training times, interpretability, and the ability to visualize the decision-making process, making them highly desirable for real-world applications.
- Deep neural networks (DNNs), particularly CNNs, excel in image classification tasks, offering automatic feature learning, spatial invariance, parameter sharing, and hierarchical representation. That is why they have been used in some existing approaches for PC diagnosis from radiographic [7,10,12] or histopathological images [8,9]. However, DNNs are not always effective for tabular data classification compared to tree-based methods. This justifies the few reported DNN-based methods for PC detection from non-radiographic data.

Our research objective is to develop machine learning approaches that can precisely predict prostate cancer without relying on expensive or invasive diagnostic procedures, such as radiography, DRE, and biopsies. In pursuit of this objective, this paper delves into the utilization of clinical biomarkers, particularly PSA levels, along with individualized data acquired through questionnaires, for the diagnosis of prostate cancer. This strategy aims to curtail the necessity for invasive procedures, consequently mitigating potential risks and discomfort for patients.

To this end, we first compiled real data from 84 PC patients. The data included PSA as a clinical biomarker, age from patient’s demographic data, in conjunction with several pieces of questionnaire-supplied information about the symptoms a patient has. Our goal was to utilize these data to develop a predictive model for prostate cancer. We then investigated and evaluated a comprehensive set of machine learning algorithms to build this predictive model. Our study included traditional classification methods, such as RFs, ANNs, and SVMs. It also covered the family of tree-based methods, such as decision trees, random forests, and gradient boosting machines, which continue to be the primary methods in the field of learning from tabular data. We also investigated employing the latest developments in deep learning methods that are particularly designed to work with tabular data, namely, TabNet [21] and TabPFN [22]. Unlike tree-based models, these deep models enable end-to-end learning for tabular data.

A key aspect of the current paper is that we investigated another, novel strategy to solve the problem at hand. This strategy was motivated by the unprecedented achievements of deep CNNs. These CNN architectures, like the sophisticated models of AlexNet, VGG, ResNet, Inception, and EfficientNet, have showcased their exceptional capability to surpass human-level performance across a diverse array of image classification tasks. They adeptly discern intricate patterns from extensive image datasets. In contrast, deep learning for tabular data classification has not been able to show similar progress. This disparity arises from the fact that tabular data lack the spatial arrangement and local correlations that CNNs are purposely built to leverage. To bridge this gap, we introduce in this paper a strategy that is based on visual representation of tabular data, which we call Tab2Visual modeling. This representation imbues the data with spatial structure and local correlations, enabling a CNN to adeptly discern and extract meaningful features crucial for classification. As such, it presents substantial potential for enhancing the performance of CNNs on tabular datasets. It also harnesses the power of CNNs and transfer learning, reducing the need for extensive labeled data by fine-tuning pre-trained models. In addition, while data in the medical fields are often limited in size, this graphical representation opens the door to employing data augmentation techniques that are more appropriate to work on images rather than on tabular data, thus increasing the size of the data. We demonstrate in this paper that this novel strategy does indeed elevate the prediction accuracy of prostate cancer.

It is important to note that we are aware of only three works in the literature that share with our Tab2Visual strategy the idea of converting non-image data into images for CNN-based classification. In the so-called DeepInsight approach [23], the data samples in the feature space are projected onto a 2-D space using t-distributed stochastic neighbor embedding (t-SNE) [24]. Then, the approach finds a minimal rectangular area on this 2-D space that includes all the projected feature points, which represents the formed image. Bazgir et al. [25] follow the same projection idea; however, t-SNE is replaced with a Bayesian multidimensional scaling to project the features onto a 2-D space. The third work, known as Image Generator for Tabular Data (IGTD) [26], forms an image by assigning each feature to an image pixel whose intensity reflects the value of the corresponding feature. Similar features are assigned to neighboring pixels while dissimilar features map to far-apart pixels. The result of IGTD is an image of the same size as the number of features in the data.

These three existing methods face challenges when dealing with datasets that have a limited number of features, as they are primarily designed for feature-rich datasets. Additionally, they encounter difficulty in generating meaningful image augmentations from the resulting images, which makes their application to smaller datasets more challenging. Compared with these methods, our proposed Tab2Visual approach offers key advantages. It can handle datasets with a small number of features. Moreover, it enables the application of powerful image augmentation techniques, which, in turn, enriches the dataset and enhances the predictive model’s ability to generalize to unseen data.

Last but not least, we investigated ensemble methods to leverage the classification accuracy. We also developed a stacking classifier that integrated the most effective methods identified in our study, demonstrating highly promising results in prostate cancer prediction from tabular data. More importantly, we deployed this predictive model online through the open-source framework, Streamlit, allowing the research community to experiment with our PC diagnostic tool.

The structure of this paper is as follows: Section 2 details the data used in this study. Section 3 describes the methods employed, while Section 4 introduces the Tab2Visual modeling approach. Section 5 reports our comprehensive experimental results, and finally, Section 6 presents our concluding remarks.

## 2. Dataset

The dataset used in our work was collected from Mansora University Hospital in Egypt. It consisted of 84 patients (45 benign prostatic hyperplasia, 39 prostatic carcinoma). The patients’ informed consent was obtained (IRB number: R21.04.1289 from IRB of the Faculty of Medicine, Mansoura University, Egypt on 9 June 2021). The dataset was composed of the patient’s age, PSA level, and personalized information about how a patient felt as taken from questionnaires. Table 1 shows a description of the dataset’s nine attributes (features). The dataset’s categorical variables were graded on a scale from zero to five, where zero indicated the absence of a condition and five signified the highest degree. The PSA and age features were used because both had already been found very relevant and useful for our classification problem by several previous works(e.g., [14]). The categorical variables were recommended by the medical members of our team to quantify the frequency and severity of symptoms as perceived by a patient. While being clinically relevant, all of them shared the advantage of being costless and rather easy for the patient to provide. For instance, in the case of the Nocturia feature, if a person woke up on four nights per week to urinate, the corresponding questionnaire response would be three(approximately half of the time). Similarly, zero denoted never, one represented seldom, two indicated less than half of the time, four signified more than half of the time, and five corresponded to always or every time.

**Figure 1:**
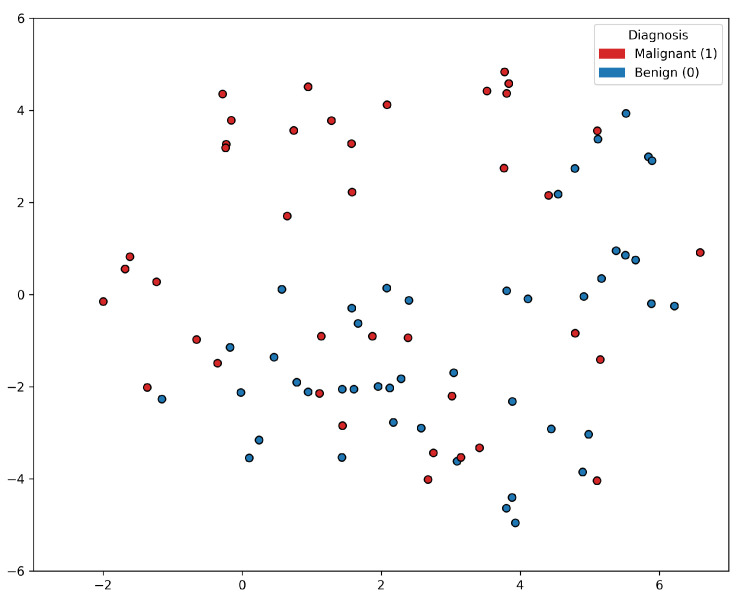
t-SNE visualization of the prostate cancer data samples. There is a lack of clear separation between the positive and negative samples.

Based on the t-SNE [24] visualization of the PC data shown in Figure 1, it appears that a group of PC positive samples forms a distinct cluster towards the upper part of the figure. On the other hand, the distribution of the negative samples is more scattered and intertwined with other positive samples. This lack of clear separation between the positive and negative samples indicates that there may not be a straightforward decision boundary that can easily distinguish between the two classes based on the features used in the t-SNE visualization. This insight suggested that the classification problem was rather complex, and linear classification models would not be sufficient for accurately predicting prostate cancer based on the selected features alone. More sophisticated, non-linear modeling techniques, such as ensemble methods or deep learning approaches, may be worth exploring to improve the classification performance.

**Figure 2:**
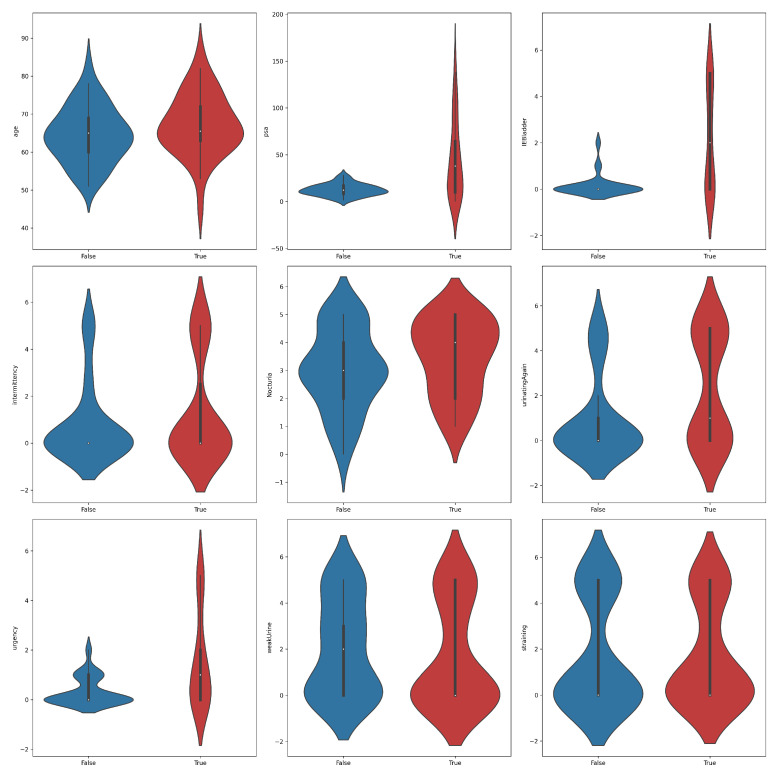
Violin plots for feature distributions in the prostate cancer dataset across the two classes, positive (true) and negative (false).

A violin plot is a statistical visualization that provides a representation of the distribution of a dataset’s numerical values, displaying both the probability density and frequency distribution. It is considered a hybrid of a box plot and a kernel density plot. The violin plots shown in Figure 2 yielded significant insights into the distribution of features across PC positive and negative samples. Notably, higher levels of PSA, IEBladder, and Urgency were associated with a higher likelihood of cancer diagnosis. Positive samples tended to exhibit narrower distributions in PSA values, suggesting a more concentrated range compared to negative samples. In terms of IEBladder and Urgency, positive samples showcased a broader range, indicative of a wider spectrum of experiences. The overlapping violin plots for other features suggested potential challenges in using them as standalone indicators of prostate cancer. Again, the findings underscored the complexity of distinguishing between positive and negative samples, emphasizing the need for a comprehensive classification approach to prostate cancer diagnosis.

**Figure 3:**
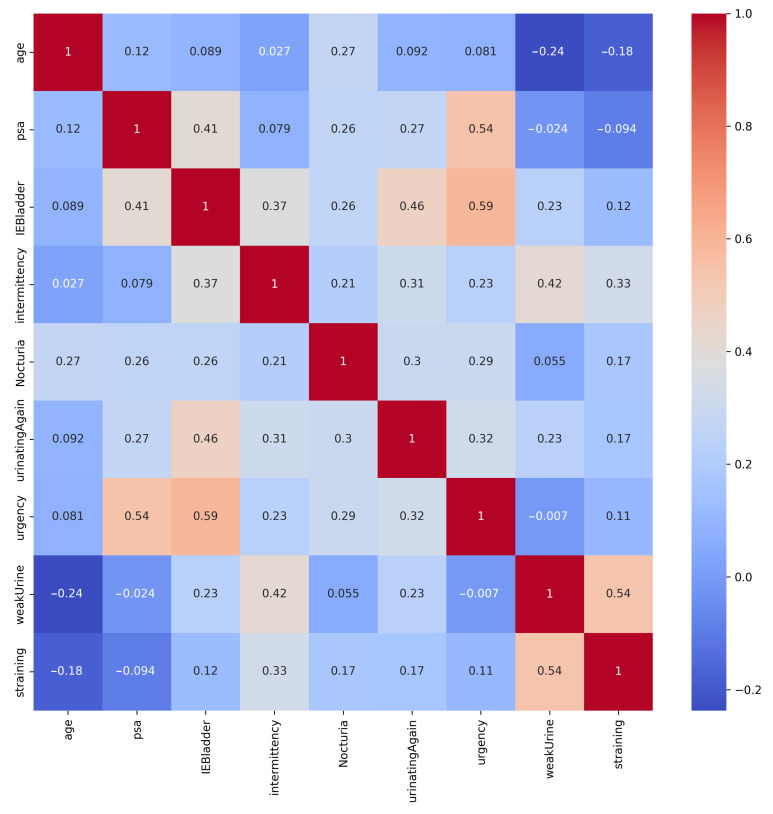
Correlation matrix of the PC dataset’s features. Strong positive correlation exists between Urgency and IEBladder, and between PSA and Urgency.

The heatmap of correlations in Figure 3 shows that the strongest positive correlation was between Urgency and IEBladder, indicating that individuals who reported urgency symptoms also tended to experience incomplete emptying of the bladder after urination. Additionally, the positive correlation between PSA and Urgency implied that individuals with elevated PSA levels could also report urgency symptoms. Furthermore, the moderately positive correlation between IEBladder and UrinatingAgain highlighted a potential association between these aspects. It is worth noting that while these correlations did provide useful associations, they did not necessarily establish causation.

## 3. Methods

In this section, we briefly describe several classification methods we investigated in our study, which was approved by the IRB of the Faculty of Medicine, Mansoura University, Egypt on 9 June 2021 (IRB number: R21.04.1289). All patients included in this study gave written informed consent to participate in this research.

### 3.1. Classical Machine Learning Methods

We investigated several classical machine learning methods. These included logistic regression (LR) [27]. LR is a versatile algorithm, particularly effective in binary classification scenarios, as it predicts target variable probabilities. Another method is support vector machines (SVMs) [28]. An SVM aims to maximize the class margin by projecting data into higher-dimensional feature spaces, enabling effective handling of non-linearly separable data. We also explored shallow artificial neural networks (ANNs) using different network architectures in terms of the number of hidden layers and their sizes.

### 3.2. Tree-Based Machine Learning Methods

Tree-based methods are commonly used in classification problems with tabular data. These methods construct a tree-like structure to determine the target variable class based on the provided features. In our study, we investigate several of these methods, including random forest (RF) [29], Extra Trees [30], extreme gradient boosting (XGBoost) [17], and Light Gradient-Boosting Machine (LightGBM) [18]. The latter is an open-source library developed by Microsoft, known for its focus on examples with large gradients, emphasizing computational efficiency and automatic feature selection. This makes LightGBM a good choice for handling large and complex datasets [18].

### 3.3. Deep Learning Methods for Tabular Data

We then explored advanced deep learning methods particularly designed to work with tabular data. The first one was the TabNet architecture by Arik and Pfister [21]. This architecture operates as a sequential attention multi-step model, with sparse instance-wise feature selection controlled by sequential attention. In addition, it quantifies feature contributions. The second one was the more recent TabPFN architecture, introduced by Hollmann et al. [22]. It is a Prior-Data Fitted Network (PFN) based on a transformer model. This architecture addresses challenges associated with tabular data training, especially on small datasets, employing progressive feature normalization and incorporating a residual connection for improved convergence speed and performance. Another advantage of TabPFN is its elimination of hyperparameter tuning, making it a convenient choice. It was shown to achieve competitive performance across various classification tasks while maintaining network compactness [22].

### 3.4. Ensemble Models

Ensemble models are powerful techniques in machine learning that combine multiple base models to improve overall performance and robustness. In our study, we utilized several types of ensemble methods: hard voting, soft voting, and stacking classifiers. Here is a general explanation of these methods.

#### 3.4.1. Voting Classifiers

The hard voting classifier makes predictions based on the majority vote from a collection of individual classifiers. Each classifier independently predicts a class label, and the final prediction is determined by the class label that receives the most votes. This method leverages the collective decision-making process to enhance accuracy and reduce the impact of individual classifier errors. Unlike hard voting, the soft-voting classifier considers the predicted probabilities of each class from all the classifiers. The final prediction is made by averaging these probabilities and selecting the class with the highest average probability. This approach benefits from the confidence levels of each classifier, often leading to more nuanced and accurate predictions.

#### 3.4.2. Stacking Classifier

The stacking classifier is an advanced ensemble method that involves multiple layers of models. The general process for stacking includes several base classifiers trained on the original dataset. Each of these classifiers generates predictions, which are then used to create a new dataset consisting of the predicted probabilities or class labels from the first-tier classifiers as features. A meta-classifier is then trained on this new dataset. The meta-classifier learns to make the final prediction based on the outputs of the first-tier classifiers and can be any machine learning model, often a simpler and robust model like a random forest or logistic regression.

### 3.5. Tab2Visual: Graphical Representation of Tabular Data

In recent years, significant strides have been made in image classification, largely due to the advent of CNNs in deep learning. CNNs learn relevant features from raw pixel values, recognize patterns regardless of position, and use shared weights for efficiency. They capture both low-level and high-level patterns, making them ideal for image classification. CNNs can also be utilized for transfer learning, reducing the need for extensive labeled data by fine-tuning pre-trained models. However, the progress in applying deep learning to tabular data classification has been comparatively gradual. This is primarily because tabular data lack the spatial structure and local correlations that CNNs are tailored to exploit.

Recognizing this discrepancy, we introduce Tab2Visual modeling, an innovative approach that bridges this gap by transforming tabular data into images. By representing tabular data as images, we introduce spatial structure and local correlations, enabling CNN to effectively learn and extract meaningful features for classification. When working with medical tabular datasets, the proposed methodology offers two immediate benefits. This graphical representation allows us to employ data augmentation techniques that are more appropriate to work on images rather than on tabular data, thus increasing the size of the data, which is a common problem with medical datasets. Second, the augmented image dataset can then be utilized to train CNNs with transfer learning, allowing the network to leverage pre-learned knowledge from large-scale image datasets.

**Figure 4:**
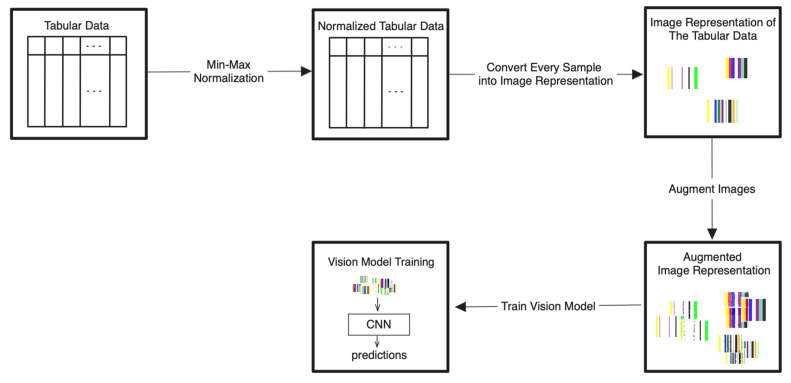
Tab2Visual modeling approach. Tabular data are normalized then encoded as images of width-varying bars. These images are then augmented and fed to CNNs.

**Figure 5:**
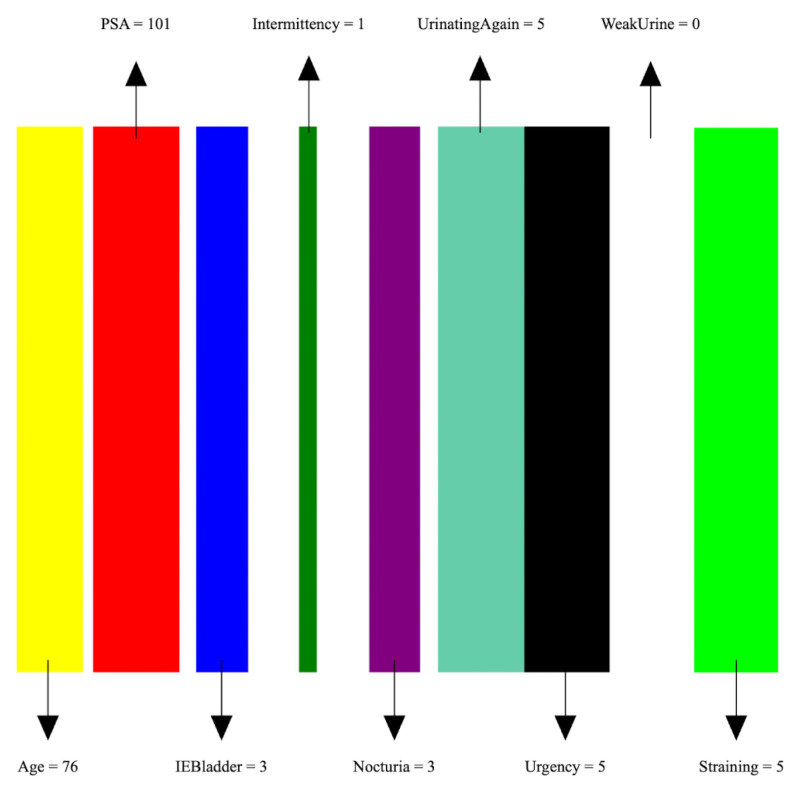
An image representation of a sample of the PC data. The image consists of 9 bars of widths proportional to feature values.

**Figure 6:**
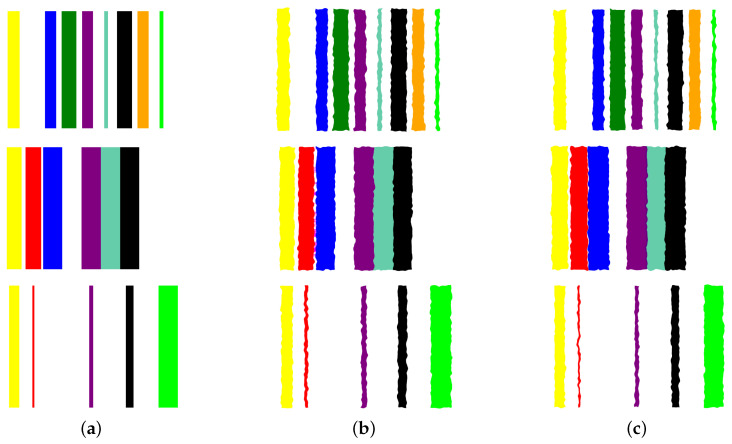
Applying augmentation techniques to the image representation of the prostate cancer data. Three samples in Column (**a**) are used to generate two image augmentations in Columns (**b**,**c**) per each.

The detailed steps for the proposed Tab2Visual modeling are illustrated in Figure 4 and explained as follows:

- Data normalization: We begin by applying min-max normalization to the tabular dataset, ensuring that each feature’s values are scaled within the range [0, 1]. This normalization process standardizes the data and facilitates consistent comparisons between features.
- Image preparation: We then create a visual representation by creating an image that consists of as many vertical bars as the number of features, *m*, in the dataset. Each bar has a maximum width of b=w/m, where *w* is the image width, thus ensuring an equal division of the image’s spatial space among the features. For example, our prostate dataset has 9 features, thus the image representation for each sample consists of 9 bars, see Figure 5.
- Feature encoding: Each feature *i* is encoded as a vertical bar in the image, such that the bar width is taken as $v_{i}\;b$, where $v_{i}$ is the *normalized* feature value. A feature with the maximum normalized value of 1 will occupy the full bar width *b*. This encoding of features as width-modulated bars visually preserves the relative magnitudes of the features in the image. Additionally, each feature is assigned a distinct color for easier visual identification.
  An example is shown in Figure 5 which shows an image representation of a sample of the prostate cancer data.
- Image augmentation: In order to increase the data size, we apply various image augmentation techniques to each image. While image mirroring, rotation, and translation are more commonly used in the literature for this purpose, we propose to use a different set of operations that better fit the semantics of the image contents. We propose to employ elastic distortion, and the morphological operations of dilation, erosion, closing, and opening, with various degrees. These operations introduce subtle variations in the bar edges and cause varying degrees of widening or shrinking effects on the image bars. This in turn helps generate novel synthetic training samples for classification model training, improving the model’s generalization to unseen data.
  More specifically, our augmentation procedure first applies elastic distortion with a random deformation strength to the image. Then, morphological dilation and erosion are performed on the image with random probabilities in a random order with a structuring element of random size. This results in an image being subjected to dilation only, erosion only, dilation followed by erosion (morphological closing), or erosion followed by dilation (morphological opening) with random varying degrees. Figure 6 illustrates three samples of the PC dataset in image representations along with some of their augmentations.
- Transfer learning with CNNs: A CNN can then be trained on the augmented dataset. To leverage the power of deep learning in image classification, we adopt transfer learning with state-of-the-art CNN models. The augmented dataset is utilized to fine-tune these pre-trained models, allowing the network to learn meaningful patterns from the transformed tabular data images.

In this paper, we relied on EfficientNet as our CNN predictive model. The EfficientNet series encompasses a range of CNN architectures renowned for achieving state-of-the-art accuracy on several classification tasks while maintaining high efficiency compared to earlier models [31]. EfficientNet V1 was introduced by Tan and Le in 2019 [31]. It introduced a composite scaling technique that uniformly adjusted network width, depth, and resolution, all guided by a predetermined set of scaling coefficients. The foundational model, EfficientNet-B0, was crafted through a combination of AutoML and the Mobile Neural Architecture Search (MNAS) framework. Subsequently, this model was upscaled to yield the successive versions, EfficientNet-B1 through B7. The “Bs” in EfficientNet refer to different variants or models within the EfficientNet architecture family. They denote the compound scaling factor that determines the model’s width, depth, and resolution. The larger the “B” value, the larger and more complex the model is.

EfficientNet V2 [32] is an enhanced iteration of the original EfficientNet series. Its development has involved a blend of training-aware neural architecture search and scaling, a dual approach that holistically optimizes the interplay between training speed and parameter efficiency. In contrast to its predecessor, EfficientNet V2 avoids utilizing depthwise convolutions and squeeze-and-excitation blocks in the initial layers. It also adopts smaller kernel sizes and contraction ratios in its mobile blocks. Moreover, it implements an upgraded progressive learning technique that contributes to both accelerated training speed and better accuracy. In several reported experiments [32,33,34,35], EfficientNet V2 is demonstrated to be effective in transfer learning tasks, achieving notable accuracy while employing fewer parameters compared to alternative models. These experiments prove it to be a fitting option for transfer learning when working with limited data. These advantages are the main motivation behind its use in our proposed Tab2Visual modeling framework.

## 4. Evaluation Metrics

To comprehensively assess the performance of our models, we employed a variety of evaluation metrics. These metrics captured different aspects of the model’s performance and ensured a robust evaluation. They are described briefly in this section.

### 4.1. Precision

Precision measures the proportion of true positive predictions among all positive predictions made by the model. It is calculated as:

$$\mathrm{Precision}=\frac{TP}{TP+FP}$$

where TP is the number of true positives and FP is the number of false positives. High precision indicates a low false positive rate, which is important in scenarios where the cost of false positives is high.

### 4.2. Recall

Recall, also known as sensitivity, measures the proportion of true positive predictions among all actual positive instances. It is calculated as:

$$\mathrm{Recall}=\frac{TP}{TP+FN}$$

where TP is the number of true positives and FN is the number of false negatives. High recall is crucial in scenarios where missing a positive instance is costly.

### 4.3. F1-Score

The F1-score is the harmonic mean of precision and recall, providing a balance between the two metrics. It is particularly useful when there is a need to balance precision and recall. It is calculated as:

$$F1-\mathrm{Score}=2\times \frac{\mathrm{Precision}\times \mathrm{Recall}}{\mathrm{Precision}+\mathrm{Recall}}$$

A higher F1-score indicates a better balance between precision and recall.

### 4.4. Area under the Curve (AUC)

The AUC of the receiver operating characteristic (ROC) curve measures the model’s ability to distinguish between positive and negative classes. The ROC curve plots the true positive rate (TPR) against the false positive rate (FPR) at various threshold settings. The AUC is calculated as:

$$\mathrm{TPR}=\frac{TP}{TP+FN}$$

$$\mathrm{FPR}=\frac{FP}{FP+TN}$$

where TP is the number of true positives, FN is the number of false negatives, FP is the number of false positives, and TN is the number of true negatives. An AUC of 1 indicates perfect classification, while an AUC of 0.5 suggests performance no better than random chance.

## 5. Experimental Results

This section reports a series of experiments using the proposed approach. Our experiments first focused on using the collected data in tabular format to predict prostate cancer employing several machine learning methods. Then, we assessed our proposed Tab2Visual approach converting tabular data into visual representations and afterwards, using CNN-based transfer learning for prostate cancer diagnosis.

### 5.1. Experiments on Tabular Representations

The methods described in Section 3 were applied to the collected dataset in its original tabular format. To assess the effectiveness of these methods, we employed leave-one-out cross-validation (LOOCV). LOOCV is a special case of K-fold cross-validation where the number of folds is the same as the number of data points. It thus fully utilizes all the available data for both training and testing (every data point is used for training and testing, but never at the same time), which is very beneficial when the data size is small or scarce, which was true in our case. Moreover, it eliminates the influence of random splitting of data. Thus, LOOCV is a nearly unbiased and reliable method of estimating a classifier’s performance [36] as long as the training and testing sets are drawn from the same distribution. However, it has the disadvantage of being more computationally expensive than K-Fold cross-validation especially for large datasets. We report the popular performance metrics of recall, precision, F1-score, and the area under the receiver operating characteristic curve (AUC) in our experiments. We carried out hyperparameter optimization using the Optuna framework [37]. Table 2 summarizes the hyperparameters related to each classifier, the parameter search range, and the best working set of parameters.

The classification results are presented in Table 3. Notably, TabPFN, LightGBM, and XGBoost models demonstrated superior performance among all methods, achieving an identical F1-Score of 0.849. TabPFN exhibited superior performance in terms of the AUC value compared to LightGBM and XGBoost, registering an AUC of 0.870. On the other hand, the performance of TabNet appeared suboptimal. This may be attributed to the small data size for accommodating the intricacies of its complex neural network architecture. In addition to being the top performer in this experiment, TabPFN exhibited the least tuning complexity; its design involved the adjustment of a single parameter, the number of ensemble configurations, in a straightforward manner.

### 5.2. Experiments on Image Representations

In the second part of our experiments, all the collected data samples were converted into images of size 640×480 pixels using the proposed Tab2Visual approach of Figure 4. This image size was determined after several preliminary experiments to balance the classification performance and computational efficiency.

Given the rather modest size of our dataset (only 84 samples), we resorted to transfer learning rather than training a CNN from scratch. Moreover, our Tab2Visual approach allowed us to apply image augmentations to increase the dataset size with different augmentation levels. To perform image augmentations, differently from the common image augmentation methods in the literature (e.g., image mirroring, rotation, and translation), we used employ elastic distortion, and the morphological operations of dilation, erosion, closing, and opening, with various degrees. More specifically, we relied in our implementation of the proposed augmentation methods on the OpenCV and Albumentations libraries. We controlled the amount of elastic distortion by the scaling factor α that controls the intensity of the displacement/deformation field and the standard deviation σ of the Gaussian filter applied to the displacement field. The higher the α, the more the image was distorted. A low σ resulted in small, localized ripple-like distortions, while a high value gave rise to larger, smoother distortions (like waves). We controlled each of the morphological dilation and erosion operations by its probability of application to the image, $P_{d}$ and $P_{e}$, respectively, as well as the size of the structuring element used. Larger structuring elements produced a more significant dilation or erosion effect. In addition, the order of performing dilation or erosion was taken randomly. The combined effect gave rise to an image being subjected to dilation only, erosion only, dilation followed by erosion (morphological closing), or erosion followed by dilation (morphological opening) with random varying degrees. Various values of the control parameters were used for generating the image augmentations: α∈[40,60], σ∈[3,5], $P_{d}\in \left[0.7,0.8\right]$, and $P_{e}\in \left[0.7,0.8\right]$. The structuring element was of size (2,5) or smaller.

We used the augmented image dataset to tune an EfficientNet V2 model. For our sake, we chose the smallest version of EfficientNet V2, more specifically, the B0 model (called herein EffNetV2_b0) pre-trained on the ImageNet-1k [38] dataset, which contains 1.2 million labeled images in 1000 categories. The reason behind choosing the smallest model was twofold; first, to mitigate overfitting due to the limited dataset size and second, to reduce the computational resources required.

It is important to stress that in our experiments, we used LOOCV on our set of 84 samples, where one sample was held out for testing, and the remaining samples were used for training. These remaining samples underwent fresh image augmentations in every iteration of the LOOCV process, preventing any potential data leakage from training to testing.

Table 4 reports the performance accuracy of the EffNetV2_b0 model for various augmentation levels. In the table, Agmnt_1 means that each sample was augmented once, thus doubling the size of the training data. In the same manner, data were enlarged 10 times for Agmnt_9. The model without any data augmentation achieved an F1-Score of 0.85 and an AUC of 0.887. All the obtained models consistently yielded AUC values higher than 0.87 for all augmentation levels. Notably, as the augmentation level increased, the performance tended to increase, reaching an AUC of 0.901 on Agmnt_7. EffNetV2_B0 on Agmnt_9 achieved an F1-Score and an AUC of 0.857 and 0.899, respectively, surpassing all the methods reported in Table 3.

We also compared the performance of the EffNetV2_b0 model against another popular CNN model, ResNet. We selected the smallest ResNet18 model for the same reasons stated before. Grid search was used for hyperparameter tuning for both CNN models, EffNetV2_B0 and ResNet18. The specifics of the hyperparameter search space and the parameters yielding the best results for each model are detailed in Table 5 without any image augmentation. Note that we trained our models for 20 epochs using the Adam optimizer and the binary log-loss function. Additionally, dropout, weight decay, and batch normalization were utilized during the training of each model to mitigate potential overfitting and improve learning dynamics. It is clear from Table 5 that while both models yielded nearly equal precision, EffNetV2_b0 offered considerably better performance on all other metrics, firmly establishing its superiority on this specific classification task.

The selection of the EffNetV2_b0 model for our study was a carefully considered decision, guided by several factors [32,33,34,35]. The EffNetV2_b0 variant is known for its balance of efficiency, performance, and effectiveness in transfer learning. This was particularly crucial given the small size of our dataset, where computational efficiency and model performance had to be optimized to avoid overfitting. EffNetV2_b0 offered a superior trade-off, ensuring that we maintained high accuracy while keeping computational demands manageable. Additionally, EffNetV2_b0’s computational efficiency was particularly beneficial for LOOCV, which is computationally intensive. The reduced complexity of the B0 variant minimized the risk of overfitting, a key concern with small datasets. Its efficiency allowed us to perform LOOCV more effectively, ensuring robust validation without excessive computational costs.

### 5.3. Discussion

**Figure 7:**
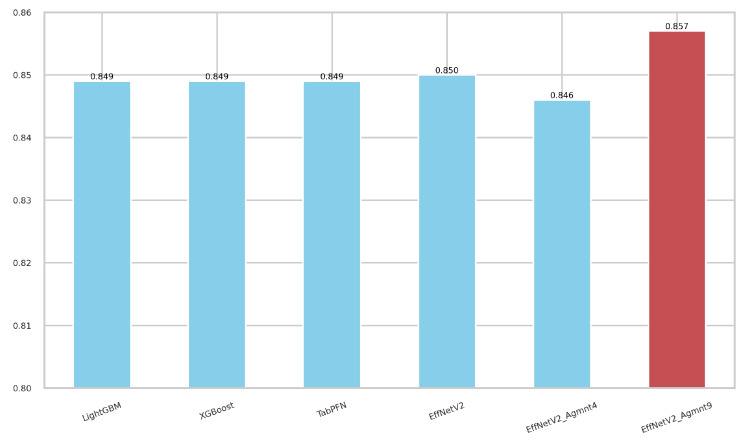
F1-scores for the best classifiers on tabular and image representations. EffNet_V2 Agmnt_9 achieved the highest F1-score of 0.857.

**Figure 8:**
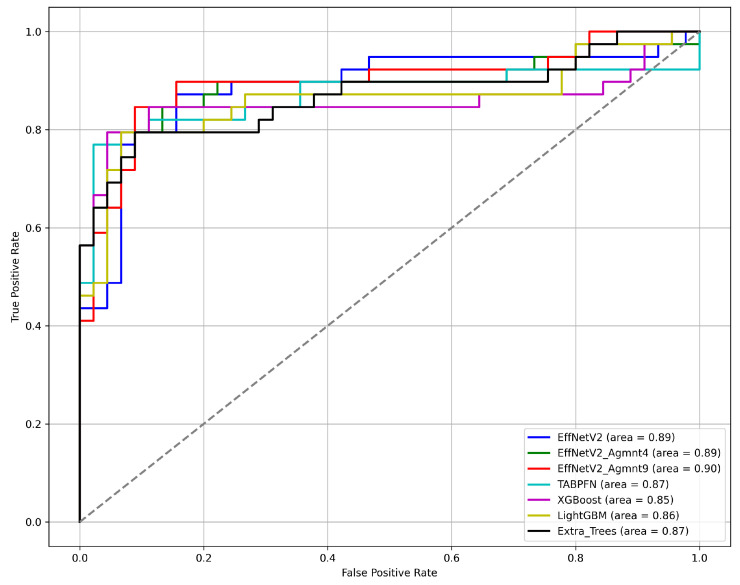
ROC curves for the best classification methods.

Figure 7 and Figure 8 summarize our findings about the most accurate predictive models in our study. The F1-scores of these models are illustrated in Figure 7. Clearly, the Tab2Visual approach improved the classification performance. Specifically, EffNetV2_B0 (Agmnt_9) achieved an F1-score of 0.857 outperforming all other classifiers. Figure 8 presents the ROC curves of all methods and their corresponding AUC values, revealing an improvement via the Tab2Visual approach. The EffNetV2_B0 model trained on the data after being modeled by the Tab2Visual approach achieved the highest AUC score of 0.901.

**Figure 9:**
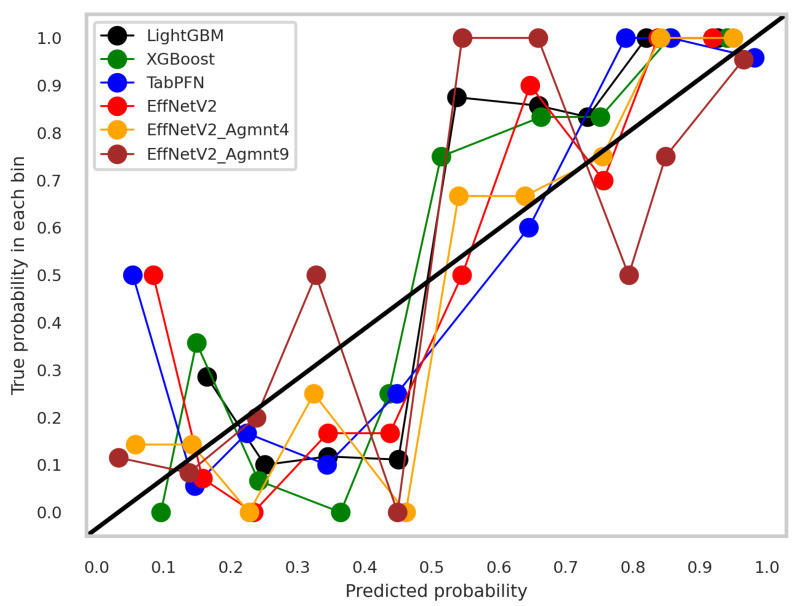
Calibration curves for the best tabular and image-based methods.

**Figure 10:**
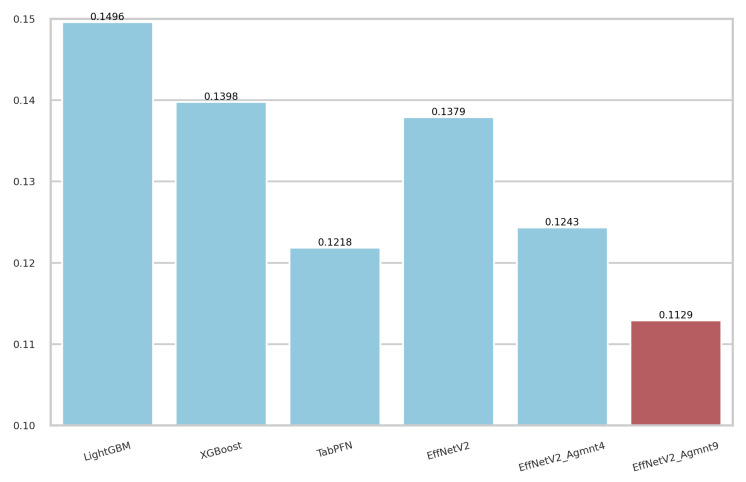
Brier scores for the best tabular and image-based methods. EffNet_V2 Agmnt_9 achieved the best score of 0.1129.

The calibration curves for the best-performing tabular and image-based methods are depicted in Figure 9. Notably, all methods exhibited a tendency to cluster around the diagonal line, signifying good calibration. To discern which classifier demonstrated superior calibration, we turned to the Brier score [39]. This metric quantifies the mean squared difference between predicted probabilities and actual outcomes, with a lower score indicating better calibration. Figure 10 presents the Brier scores for each method. Clearly, EffNetV2_B0 (Agmnt_9) achieved the highest level of calibration, attaining a Brier score of 0.1129.

All these results underscored the performance gain by the proposed Tab2Visual approach, emphasizing the potential benefits of integrating image representation in the classification task.

**Figure 11:**
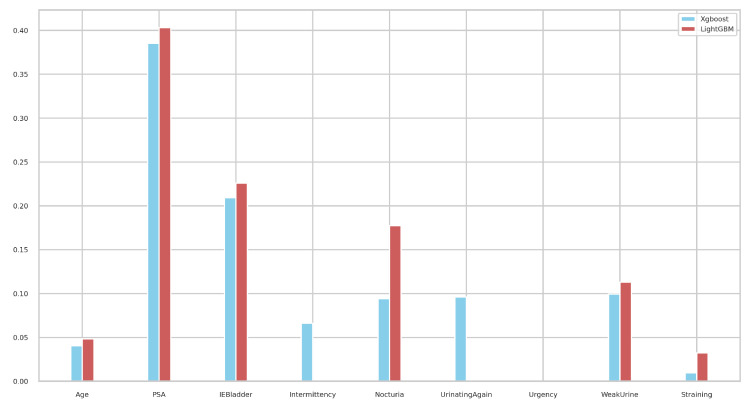
Feature importance values of the tree-based XGBoost and LightGBM classifiers.

**Figure 12:**
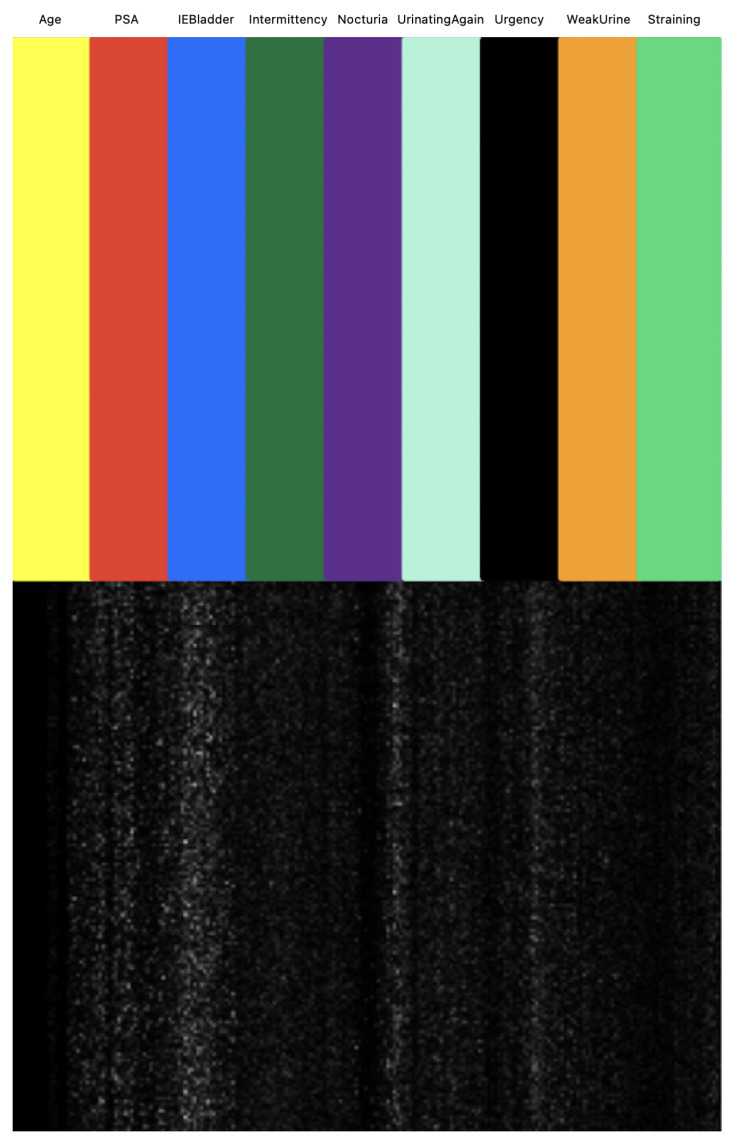
Approximation of Tab2Visual feature importance values using Gradient Explainer. The density of white points indicates areas the model focuses on to make a decision.

We next investigated the feature importance values for the tree-based classifiers (XGBoost and LightGBM). Figure 11 illustrates the feature importance values as identified by tree-based classifiers. Notably, PSA emerged as the most significant in both models, followed by IEBladder, which also showed considerable importance. In Figure 12, we explored the feature importance for the Tab2Visual model (particulary, EffNetV2 with 9 augmentations), using Gradient Explainer [40], a tool for interpreting CNN predictions and helping in approximating the feature importance values of the model. The density of white points indicates areas the model focused on to make a decision. Consistent with the tree-based classifiers, PSA and IEBladder feature areas showed the highest intensity of white pixels, underlining their critical role in the prediction. The medium intensity of white pixels in areas corresponding to Nocturia, Intermittency, and Urgency suggested these features also contributed to the decision-making process. However, regions representing Age and Straining had minimal or no white pixel intensity, implying that these features were less important.

It is also of interest to comment on the time performance of all classification methods. All methods were implemented on a workstation equipped with an Intel Core i9 CPU running at 3 MHz, featuring 18 cores, 256 GB of RAM, and an NVidia Quadro RTX 5000 GPU with 12 GB of dedicated memory. Table 6 reports the training times for all models in our study, derived from the entire LOOCV process involving 84 samples. As anticipated, the neural network-based methods showed longer training times, with EffNetV2_B0 exhibiting the longest time owing to its inherently more complex CNN architecture. However, when it came to inference time, which measures the time duration for making a prediction on a single sample, all models demonstrated fast response times. This makes them well suited for real-time operation in computer-aided diagnosis (CAD) systems. Of particular interest, according to Table 6, the EffNetV2_B0 model demonstrated an inference time of 21ms, significantly better than its training time.

### 5.4. Ensemble Models

Our goal here was to investigate leveraging the classification performance further by constructing an ensemble model with enhanced predictive capability. To this end, we selected the most effective classifiers from our study, namely, TabPFN, LightGBM, EffNetV2_B0 (No Agmnt), EffNetV2_B0 (Agmnt_4), and EffNetV2_B0 (Agmnt_9), to build both hard- and soft-voting classifiers. The hard-voting classifier determined predictions by majority voting from the individual classifiers, while the soft-voting classifier calculated the average predicted probability across all classifiers.

**Figure 13:**
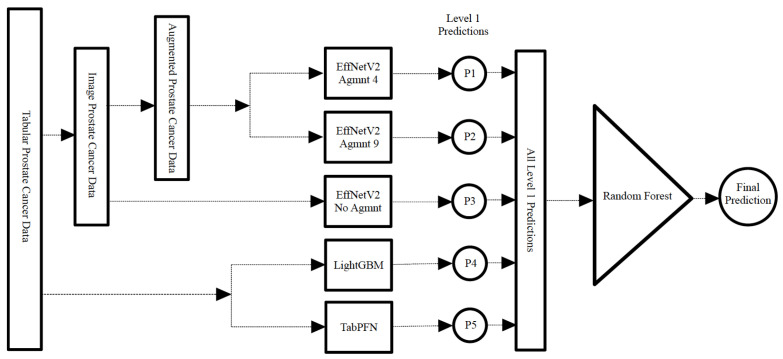
Construction of the stacking classifier with the best-performing models in level 1 and a random forest meta classifier.

Additionally, we designed a stacking classifier, illustrated in Figure 13, comprising the aforementioned top-performing classifiers followed by a random forest meta-classifier. In this approach, the first-tier classifiers were trained on the entire dataset, with one sample held out for testing. The predictions from these first-tier classifiers were then aggregated to train the meta-classifier. This resulting stacking classifier was subsequently applied to the held-out test sample. This process was iterated for all samples in the dataset as typically done in LOOCV.

**Figure 14:**
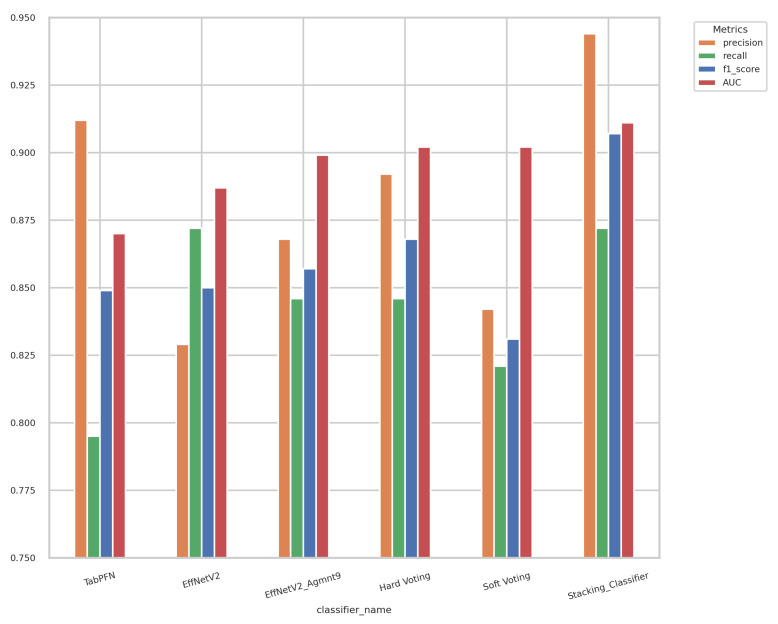
Results of ensemble models and best-performing individual classifiers. The stacking classifier achieved the highest F1-score and AUC value of 0.907 and 0.911, respectively.

Displayed in Figure 14 are the outcomes of the hard-voting, soft-voting, and stacking classifiers in comparison with the performance of the top-performing individual classifier. The stacking classifier demonstrated a notable enhancement surpassing all other classifiers, attaining an F1-score of 0.907 and an AUC of 0.911.

Our proposed, top-performing model, the stacking classifier, was deployed in the public domain as an Internet-accessible web application. This user-friendly tool is powered by Streamlit, an open-source framework designed for the swift development and sharing of machine learning and data science web applications. Our goal here is to allow the research community to experiment with our PC screening tool. The interested reader can experience this tool by visiting the web address: https://prostate-cancer-diagnosis.streamlit.app/ (last accessed 20 June 2024).

### 5.5. Overfitting Mitigation and Regularization

To ensure our Tab2Visual approach avoided overfitting, we employed several strategies. We leveraged transfer learning by using pre-trained convolutional neural network (CNN) models, specifically EfficientNet V2 (the B0 model). The B0 model, being the smallest in the EfficientNet family, helped in avoiding model complexity and mitigating overfitting. During fine-tuning, we froze all the EfficientNet model weights, allowing the model to benefit from the generalization capabilities of models trained on large-scale datasets. Additionally, we included batch normalization, strong weight decay, and dropout for regularization during the fine-tuning process to prevent the model from relying too heavily on specific weights or neurons. Data augmentation played a crucial role in enhancing the model’s robustness. We applied various image augmentation techniques such as elastic distortion and morphological operations (dilation, erosion, closing, and opening) to introduce subtle variations in the training data, thus improving the model’s ability to generalize to unseen data.

For tabular data methods, we included ensemble tree-based methods such as random forests and gradient boosting. Ensemble methods combine the predictions of multiple models to improve robustness and reduce overfitting. We also fine-tuned the regularization parameters in these methods to control model complexity. Furthermore, we built an ensemble of the best-performing methods, carefully selecting diverse base models that handled the data differently. This diversity in model approaches enhanced performance and reduced the effect of overfitting by balancing out individual model shortcomings, ensuring robustness and generalizability in the final predictions.

## 6. Conclusions

In this paper, we presented a comprehensive study aimed at developing an accurate and non-invasive diagnostic tool for prostate cancer. Leveraging clinical biomarkers, like PSA levels, and personalized patient data obtained through questionnaires, our approach provided a viable alternative to invasive procedures, such as biopsies, and costly MRI scans. To develop this tool, we conducted an extensive evaluation of various classification models, spanning from traditional to tree-based classifiers, and explored advanced deep learning architectures tailored for tabular data. Moreover, we introduced the Tab2Visual modeling approach, which transformed tabular data into image representations, demonstrating superior performance compared to other methodologies. More specifically, employing EfficientNet V2 on augmented image representations, we achieved an F1-score and AUC of 0.857 and 0.899, respectively.

Furthermore, we investigated ensemble methods to leverage classification accuracy. We developed a stacking classifier, incorporating the top-performing individual classifiers of our study in the initial layer, complemented by a random forest meta-classifier. This ensemble model excelled in distinguishing benign and malignant cancer, yielding an F1-score of 0.907 and an AUC of 0.911. These results strongly indicated that our proposed approach showed great promise as a cost-effective alternative to current, more invasive, and costly diagnostic procedures for prostate cancer.

We believe that this paper makes the following contributions:

- Addressing a novel research problem of predicting prostate cancer from PSA levels and individualized questionnaires without relying on expensive or invasive diagnostic procedures.
- Collection of clinical data from 84 patients, including PSA levels and personalized information through questionnaires, to develop this predictive model.
- Comprehensive assessment and comparison of various machine learning techniques, including traditional, tree-based approaches, and advanced tabular deep neural network architectures (TabNet and TabPFN) for accurate and cost-effective prostate cancer prediction.
- Introduction of Tab2Visual modeling, a novel approach that converts tabular samples into image representations, enabling powerful and novel image augmentations to increase the dataset size and enhance the model’s ability to generalize to unseen data. It allows the use of CNNs with transfer learning, resulting in higher prediction accuracy.
- Development of a stacked classifier integrating the most effective methods identified in the study, demonstrating highly promising performance in prostate cancer prediction.
- Sharing our best predictive model with the research community through its deployment on the Internet as a web application using the Streamlit open-source framework.

## 7. Limitations and Future Work

We strongly believe that the proposed Tab2Visual modeling approach holds great potential for significantly enhancing the performance of deep learning models on tabular datasets in general. However, we do realize that the proposed approach in this paper has some limitations that require further investigation:

- The use of deep learning models such as EfficientNet V2 involves significant computational overhead in terms of training time and hardware resources. To address this, we are optimizing our models for efficiency by exploring lightweight architectures and pruning techniques that maintain high performance while reducing computational demands.
- The Tab2Visual modeling converts tabular data into an image of width-modulated bars arranged in a single row. When dealing with datasets with many features, each feature is allocated a narrower bar in the image, which can make it challenging for the model to distinguish between features. To address this, we are exploring alternative layouts for the visual representation, such as arranging the bars in multiple rows or using other geometric shapes that can better accommodate a higher number of features without compromising clarity.
- Transforming tabular data into visual representations can sometimes lead to a loss of interpretability, especially for domain experts accustomed to traditional tabular formats. To improve interpretability, we are developing tools and interfaces that allow users to seamlessly switch between the tabular and visual representations and are working on enhancing the explainability of our models by integrating visualization techniques that highlight the most important features and their contributions to the model’s predictions.
- The dataset used in this study is relatively small, comprising only 84 samples, which may affect the generalizability of the model. We are actively seeking to expand our dataset by collaborating with additional medical institutions and incorporating data from different demographics and geographical locations, which will enable more robust training and validation of our model.

As part of our ongoing research endeavors, we are currently focusing on training and validating our approach on larger datasets and investigating other image augmentation methods for the proposed Tab2Visual approach. This pursuit is expected to yield improved accuracy for the proposed PC diagnostic tool.

## Figures and Tables

**Table 1:** Prostate cancer dataset description.

Feature Name	Description	Feature Type
PSA	Prostate-specific antigen value	Continuous
AGE	Age	Continuous
IEBladder	Frequency of feeling that the bladder is not completely empty after urination	Categorical
UrinateAgain	Frequency of urinating again in less than 2 h	Categorical
Intermittency	Frequency of the urine stream cut-off during urination	Categorical
Urgency	Frequency of difficulty to delay urination	Categorical
Nocturia	Frequency of getting up at night to urinate	Categorical
Straining	Frequency of difficulty to start urination	Categorical
WeakUrine	Frequency of feeling the weakness of the urine stream	Categorical

**Table 2:** Hyperparameter tuning for tabular classification methods.

Method	Hyperparameter	Search Space	Best Value
Logistic regression	penalty	[l1, l2]	l1
	C	0.01 to 1	0.248
SVM	C	0.001 to 100	3
	kernel	[‘linear’, ‘poly’, ‘rbf’]	‘rbf’
	gamma	0.001 to 100	0.1
Random forest	n_estimators	1 to 500	46
	max_depth	1 to 40	8
	min_samples_split	2 to 14	1
	min_samples_leaf	1 to 14	1
	max_features	[‘auto’, ‘sqrt’, ‘log2’]	‘log2’
Extra Trees	n_estimators	1 to 500	96
	max_depth	1 to 40	10
	min_samples_split	2 to 14	51
	min_samples_leaf	1 to 14	1
	max_features	[‘auto’, ‘sqrt’, ‘log2’]	‘sqrt’
XGBoost	n_estimators	1 to 500	18
	max_depth	1 to 40	3
	gamma	0 to 5	4.2854
	learning_rate	0.01 to 1	0.307
	reg_alpha	0 to 2	1.779
	reg_lambda	0 to 2	0.439
	subsample	0.5 to 1	0.996
	colsample_bytree	0.5 to 1	0.733
LightGBM	n_estimators	1 to 500	18
	max_depth	1 to 20	6
	num_leaves	2 to 256	98
	learning_rate	0.01 to 1	0.358
	reg_alpha	0 to 2	1.699
	reg_lambda	0 to 2	0.872
	subsample	0.5 to 1	0.698
	colsample_bytree	0.5 to 1	0.762
CatBoost	iterations	50 to 300	72
	learning_rate	0.01 to 0.3	0.3720
	depth	2 to 12	2
	l2_leaf_reg	1 to 10	8.712
Shallow ANN	n_hidden_layers	[1, 3]	2
	n_neurons_hidden_layer	1 to 256	32–8
	learning_rate	0.0001 to 0.1	0.0501
	batch_size	[16, 32, 64, 128]	16
	weight_decay	0.00001 to 0.01	0.001
	drop_prob	0.1 to 0.7	0.5
TabNet	batch_size	[8, 16, 32, 64]	16
	mask_type	[‘entmax’, ‘sparsemax’]	‘sparsemax’
	n_d	8 to 64 (step 4)	8
	n_a	8 to 64 (step 4)	8
	n_steps	1 to 8 (step 1)	5
	gamma	1.0 to 1.4 (step 0.2)	1.2
	n_shared	1 to 3	2
	lambda_sparse	0.0001 to 1	0.000106
	patienceScheduler	3 to 10	6
	learning_rate	0.001 to 1	0.02
TabPFN	n_ensemble_configurations	[1, 32]	3

**Table 3:** Classification results by various classification methods on tabular representations.

Classifier	Precision	Recall	F1-Score	AUC
LR	0.931	0.692	0.825	0.836
SVM	0.903	0.718	0.800	0.816
LightGBM	0.912	0.795	0.849	0.861
XGBoost	0.912	0.795	0.849	0.854
Random forest	0.815	0.795	0.805	0.862
ExtraTrees	0.886	0.795	0.838	0.869
ANN	0.816	0.795	0.805	0.856
TabNet	0.811	0.769	0.789	0.859
TabPFN	0.912	0.795	0.849	0.870

**Table 4:** Performance of EffNetV2_B0 on image representations with varying augmentation levels.

Augmentation	Training Size	Precision	Recall	F1-Score	AUC
No Agmnt	83	0.829	0.872	0.850	0.887
Agmnt_1	166	0.864	0.820	0.842	0.870
Agmnt_2	249	0.833	0.769	0.800	0.877
Agmnt_3	332	0.800	0.821	0.810	0.872
Agmnt_4	415	0.846	0.846	0.846	0.886
Agmnt_5	498	0.861	0.794	0.827	0.875
Agmnt_6	581	0.886	0.795	0.839	0.873
Agmnt_7	664	0.886	0.795	0.839	0.901
Agmnt_8	747	0.825	0.846	0.835	0.897
Agmnt_9	830	0.868	0.846	0.857	0.899

**Table 5:** Hyperparameter tuning for classification methods for image representations.

Method	Parameter	Search Space	Best Value	Method Evaluation
ResNet18	n_hidden_layers	{0, 1, 2}	0	Precision: 0.806 Recall: 0.872 F1-Score: 0.850 AUC: 0.887
	n_neurons_layer	{32, 64, 128, 256}	No hidden layer	
	learning_rate	[0.000001, 1]	0.001	
	weight_decay	[0.0001, 0.5]	0.01	
	drop_out_probability	[0.1, 0.7]	0.5	
EffNetV2_B0	n_hidden_layers	{0, 1, 2}	0	Precision: 0.806 Recall: 0.872 F1-Score: 0.850 AUC: 0.887
	n_neurons_layer	{32, 64, 128, 256}	No hidden layer	
	learning_rate	[0.000001, 1]	0.001	
	weight_decay	[0.0001, 0.5]	0.01	
	drop_out_probability	[0.1, 0.7]	0.5	

**Table 6:** Time performance for the various classifiers investigated in our study.

Classifier	Training Time (s)	Inference Time (ms)
LR	2.3	1.2
SVM	4.4	0.2
Random forest	18	1.7
ExtraTrees	9	1
LightGBM	6.8	1.5
XGBoost	11.4	2
ANN	67	10
TabNet	544	7
TabPFN	21	101
EffNetV2_B0	3780	21

## Data Availability

Data are available upon reasonable request to the corresponding author.
